# Measurable Prediction for the Single Patient and the Results of Large Double Blind Controlled Randomized Trials

**DOI:** 10.1371/journal.pone.0001909

**Published:** 2008-04-02

**Authors:** Cathy M. Helgason, Thomas H. Jobe

**Affiliations:** 1 Department of Neurology, University of Illinois College of Medicine at Chicago, Chicago, Illinois, United States of America; 2 Department of Psychiatry, University of Illinois College of Medicine at Chicago, Chicago, Illinois, United States of America; Memorial Sloan-Kettering Cancer Center, United States of America

## Abstract

**Background:**

It has been shown that the clinical state of one patient can be represented by known measured variables of interest, each of which then form the element of a fuzzy set as point in the unit hypercube. We hypothesized that precise comparison of a single patient with the average patient of a large double blind controlled randomized study is possible using fuzzy theory.

**Methods/Principle Findings:**

The sets as points unit hypercube geometry allows fuzzy subsethood to define in measures of fuzzy cardinality different conditions, similarity and comparison between fuzzy sets. A fuzzy measure of prediction is defined from fuzzy measures of similarity and comparison. It is a measure of the degree to which fuzzy set A is similar to fuzzy set B when different conditions are taken into account and removed from the comparison. When represented as a fuzzy set as point in the unit hypercube, a clinical patient can be compared to an average patient of a large group study in a precise manner. This comparison is expressed by the fuzzy prediction measure. This measure in itself is not a probability. Once thus precisely matched to the average patient of a large group study, risk reduction is calculated by multiplying the measured similarity of the clinical patient to the risk of the average trial patient.

**Conclusion/Significance:**

Otherwise not precisely translatable to the single case, the result of group statistics can be applied to the single case through the use of fuzzy subsethood and measured in fuzzy cardinality. This measure is an alternative to a Bayesian or other probability based statistical approach.

## Introduction

Physicians make diagnostic and treatment decisions based on their perception of how scientific evidence matches the clinical patient in question. This perception and decision are inscrutable, often requiring different degrees of expertise. Before the advent of The Generalized Theory of Uncertainty (GTU) developed by Lotfi Zadeh, precise representation and calculation with perceptions expressed in natural language had been impossible. [1] Precise presentation of the way in which physicians perceive a patient's condition and make clinical decisions requires representation of the human cognitive skills of graduation and granulation in such form as computation might take place. This representation is satisfied by the tools of fuzzy logic and GTU. [1] Information can now be expressed in non statistical form. [1] This is quite different from information gained from large double blind randomized clinical trails which is statistical in form, based in bivalent Aristotelian logic and probability theory.

Fuzzy theory admits everything to be present to a degree. Thus, when considering the physiologic or pathologic elements of a patient, those elements can each be given a value in the unit interval without constraint on nor being constrained by the value of the others in order to form the element of a fuzzy set. [2] Numerical valuation for any element of clinical interest is achieved through laboratory measurement and normalization or through expert assignment. The advantage to representing the patient's clinical state defined by the elements in a fuzzy set is that no value of any element is constrained by those of any other. This property of fuzzy logic also allows overlap of value assignment in a fuzzy graph or granulation.

We have described how the fuzzy “sets as points” representation of a patient's clinical state in the unit hypercube allows for the visualization, demonstration and ultimate measurement of the different conditions of each fuzzy set as point. [3], [4] Those conditions are by their nature, symmetries of fuzzy cardinality of all the other fuzzy sets as points in the unit hypercube. A measure for different degrees of symmetry breaking and restoration of new symmetry of conditions has been defined by us and called “K”. [4] This measure provides a means to precisely represent the difference in conditions of any two patients.

Scientific medicine demands that in order for two patients to be compared their conditions must be no different. This is the same as the requirement that at each repetition of an experiment conditions remain unchanged. For this reason scientific medicine relies on statistics based in probability theory. In this study we show how one unique patient can be compared to the average patient of a large double blind controlled randomized trial in a precise and measurable fashion using fuzzy theory. This method is an alternative to the Bayesian approach of “Evidence-Based Medicine” founded in probability theory. It is by its very nature of foundation in fuzzy theory different from other probability based statistical approaches.

## Results

The calculation of risk reduction for a clinical patient presenting to the physician, B, compared to the risk reduction for the average study patient, A, of a chosen large double randomized controlled clinical trial, the Caprie Study, is given by the following using the clinical information provided in the materials and methods section of this paper:

A = {0.5, 0.5} and B = {0.7, 0.6}, where fuzzy sets as points A and B represent patient A and patient B, and fuzzy set elements are time since stroke and time on study drug normalized to fall within the unit interval.
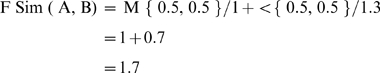



K = 1.49, where K is the measure of breaking symmetry of conditions
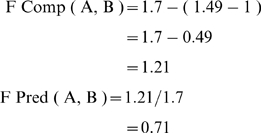



The overall risk reduction for vascular mortality and ischemic stroke in the Caprie study in favor of clopidogrel compared to aspirin was 8.7%. Our patient has benefited from clopidogrel by 6.18% as measured by F Pred (A, B) for our patient compared to the average patient of the Caprie study, or 0.71×8.7 = 6.18%. Results of the average patient form a large double blind controlled randomized trial, the Caprie study, can be measurably adapted to the single patient at the bedside to calculate the risk reduction for clopidogrel. The hypothesis that fuzzy measures can achieve this is proven true. Our hypothetical patient has had a 6.18% risk reduction for vascular mortality and ischemic stroke compared to if he/she had been on aspirin.

Other results of the Caprie study can also be applied to this patient. The risk reduction for the average Caprie study patient for all cause mortality and all cause strokes was 6.9% for those patients taking clopidogrel. For our clinical patient his/her overall risk reduction for these parameters is 0.71×6.9, or 4.90%. Now had the patient inquired, well what are my “chances” of having an ischemic stroke if I am on aspirin? The primary analysis of outcome in the Caprie study showed that 4.8% of patients on aspirin had an ischemic stroke. We use our same numbers for comparison of our patient to the average patient of the trial, because these do not change, but now we multiply our fuzzy prediction measure by 4.8% and get 0.71×4.8 to get 3.4% and if on clopidogrel 0.71×4.6 to get 3.27%; of having a myocardial infarction on aspirin 0.71×3.5% or 2.49% for aspirin versus 0.71×2.06% or 1.46% if on clopidogrel.

In this study we did not use the fuzzy entropy measures of similarity and symmetry because the problem at hand, to compare the outcomes of the average patient in the Caprie study to our hypothetical bedside patient did not require so.

## Discussion

We defined several *fuzzy* measures for comparison of individual fuzzy “sets as points” in the unit hypercube. [5], [7] The geometric structure of the unit hypercube and sets as points representation was chosen in order to develop these measures from the *fuzzy* subsethood theorem, and the *fuzzy* entropy theorem, measured in *fuzzy* cardinality. [7] Of particular interest was the ability to compare fuzzy sets as points not only by the values of their elements, but as to their different conditions. These conditions are all the surrounding fuzzy sets as points in the unit hypercube, each of which has a known fuzzy cardinality. A fuzzy measure of breaking of symmetry of conditions, called “K”, defined from fuzzy subsethood and measured in fuzzy cardinality was employed for this purpose. [4] A demonstration of K exists when two fuzzy sets are exchanged in the unit hypercube. If considered a ball of certain size defined by fuzzy cardinality, the exchange of two such fuzzy sets as points disturbs the position of all the others, if the exchange takes place between two fuzzy sets as points of different size. Without the fuzzy sets as points geometry in the unit hypercube, the changing symmetry of conditions would not be demonstrable in this fashion. [3] We have elsewhere demonstrated the symmetry breaking property of K using continuous cellular automata where K is the multiplicative factor and 1/K the initial seed. [8]

In this paper, the reason for comparing two fuzzy sets as points in the unit hypercube was to develop a means of prediction for the single case. Herein, the single case is a patient. We focused on the problem posed to the medical physician in opining diagnosis and treatment when such decisions are to be “scientifically” based. The problem is two fold. On the one hand, physician decisions based on expertise are considered vague and ambiguous, and for this reason *un*scientific. On the other hand, in response to this fact, medical science has adopted the stance that physician decisions should be based on the results of large double blind controlled randomized clinical trials.[9] This is because these trials are based in probability theory and promise certainty in their results. This certainty rests on the property of uniform conditions. These uniform conditions can be demonstrated in the unit hypercube within the probability space. [5] The physician however must make his match of patient to average patient of the clinical trial all while considering the different conditions of the two.

We have previously noted that the decision of how to apply and adapt the results of these trials to the individual clinical patient depends on the physician's ability to match the trial results to that patient. This matching process requires cognitive skill that in itself is inscrutable because no individual patient has the same conditions or context as those possessed by the average patient of the large group trial. Conditions or context refer to all those unknown unmeasured elements that might affect the clinical behavior or outcome of the patient. These conditions are assumed to be uniform in the large double blind randomized clinical trial, but in reality can never all be accounted for in any population of different individuals. This is demonstrable in the unit hypercube outside of the probability space where known measured element values of each fuzzy set as point are not constrained by their sum always being certain. Evidence based medicine can use a pooled analysis based on data from clinical trials and applies the model to the patient. This results in a probability measure of estimated risks for the single patient based on his known measured elements. [9] Different unknown conditions remain unaccounted for in their role of how accurate the prediction based on probabilities turns out to be for that patient.

In general, the process of diagnosis and treatment requires the cognitive skills of graduation and granulation, both capable of precise representation within the GTU. The fuzzy measure of breaking of symmetry of conditions allows for a quantitative representation of different conditions of different patients, thus allowing an exact match to be measured between any patient and the average patient of a clinical trial. We propose that this process can be used as a step in the process of computing measured risk for the single patient based on results of clinical trials. This step takes into account the difference in the degree of presence of each clinically relevant factor as well their different conditions.

While Bayes' theorem is one basis for making scientific clinical decisions at the bedside, it does not give the same measure as we have developed in this paper with the fuzzy prediction measure. The Bayesian approach allows the physician to know the probability that a study applies to a given clinical patient who has certain known measured elements, but this is followed by an intuitive stance on the part of the physician in applying the results of the clinical trial to the unique patient facing him. [7] This is because that unique patient has his own personal unique context or conditions, which are different from those of the average patient of the clinical trial. The fuzzy prediction measure used in our study gives an exact translation of study results to the individual patient, with no room for chance in that match to the average patient of the trial.

We proposed that instead of relying on probability theory and statistical information, fuzzy theory within the general context of GTU may solve the problems posed in representing bedside clinical information and physician decision. [1] The standard fashion of applying the results of a large clinical trial to an individual patient requires building a statistical model using trial data and applying the model to the patient according to known measured risk factors of that patient. [9] This method, while elegant, does not account for the different conditions of the patient that are not accounted for by known measured variables. It assumes that unknown factors are present, but somehow their effect on the known measured variables cancels out. It also provides information in the form of a probability. Because it is founded on probabilities it is fundamentally different from the fuzzy prediction measure which does not rely on bivalent Aristotelian logic nor assume uniform conditions in the comparison between the clinical patient and those of the trial.

We have shown in a previous paper that measures of comparison for two single patients can easily be defined using fuzzy subsethood and entropy. [5] This is because fuzzy theory is able to account in a measurable way for different conditions of different individual patients. These conditions can be demonstrated in the unit hypercube as having a symmetry that can be broken and restored to a certain degree when one fuzzy set as point is compared or transforms to another. In the instance of following one patient over time at different clinical states, each state represented by known measured variables of interest and as fuzzy sets as points in the unit hypercube can be said to transform to another. This transformation or comparison between fuzzy sets as points in the unit hypercube involves a dynamic where symmetry breaking and restoration to a degree of conditions and a subsethood relation of known measured variables characterizes the action.

In this study we are able to show that the fundamental concept of fuzzy theory, subsethood, and its primary measure space, fuzzy cardinality as instantiated in the fuzzy unit hypercube can be employed to give a precise and measurable match of the single clinical patient to the average patient of any large double blind randomized clinical trial. This ability has the potential to render precise physician decisions at the bedside and gives new relevance to the results of clinical trials. We intend to test this hypothesis by taking the results of a large double blind randomized controlled clinical trial, the AAASPS ( African American Antiplatelet Stroke Prevention Study) trial, and compare each patient in that trial to the average patient of the trial using the fuzzy prediction measure. This comparison will test the predictive capacity of the fuzzy prediction measure for each patient in the trial because the outcome of each patient is already known from the trial results. [10].

## Materials and Methods

Medical science has relied on probability theory and the large double blind controlled randomized clinical trial in order to guarantee certainty in the calculation of the relation of known measured variables without interference of different conditions. We have shown that different conditions of different patients can be measured by the symmetry breaking measure “K” as two fuzzy sets as points in a unit hypercube, representing two different patients A and B, are compared. [4]

The measure K and the measure of fuzzy similarity of two fuzzy sets as points in the unit hypercube (F Sim (A, B)) are derived from fuzzy subsethood and the primary measure space of fuzzy theory fuzzy cardinality. This measure allows two sets as points in a unit hypercube to be compared precisely while accounting for different conditions.

We hypothesized that the fuzzy measure of prediction ( F Pred (A,B)) defined by us using fuzzy subsethood and measured in fuzzy cardinality can be used to precisely compare an individual clinical patient to the average patient of a large double blind randomized controlled clinical trial. [5]

Every large double blind randomized controlled clinical study has the concept of an average patient. The concept of average enforces symmetry of conditions in a statistical sense. We take the results of such a large group clinical study call the Caprie Study. The average patient in this study took an antiplatelet agent clopidogrel 75 mg daily or aspirin 325 mg daily for a period of 1.6 years. Randomization required having suffered an ischemic stroke within 6 months of randomization. When faced with the clinical patient, the physician wants to be able to predict for any patient taking clopidogrel under similar circumstances the effect of clopidogrel or aspirin on that patient's outcome.

The overall risk reduction for vascular mortality and ischemic stroke in the Caprie study was in favor of clopidogrel by 8.7%. [6] We take the example of a hypothetical patient. The patient in question had a stroke 9 months before he started his clopidogrel and has now been taking it for 2 years. How much ash clopidogrel reduced his risk compared to if he had been on aspirin? For the purpose of testing our hypothesis we use the example of this hypothetical patient and assume that time from stroke onset was 6 months for the average patient in the Caprie study.

The average patient A of the Caprie study and our patient B are compared using our definition of fuzzy similarity and fuzzy comparison, F Sim (A, B) and F Comp (A, B). The fuzzy measure of prediction tells the physician how much A is equal to B given their different conditions. It is expressed by F Comp (A, B)/F Sim (A, B). Given the known elements of time from stroke and time on medication, we can represent the average patient from the Caprie study and the clinical patient presenting to the physician by these elements where each patient is represented as a fuzzy set as point in the unit hypercube. After normalization of time since stroke by 12 (months) and time on study drug by 3 (years) each element falls within the unit interval. The average patient from the Caprie study can be represented as fuzzy set A { 0.5,0.5 } and the clinical patient B { 0.7,0.6 }.

The F Sim ( A , B ) and F Comp ( A, B ) measures are calculated using the definition of F Sim (A,B ) = S ( A,B )+S ( B, A ) , where S stands for fuzzy subsethood . Fuzzy subsethood of A in B is the degree to which fuzzy set as point A belongs to fuzzy set as point B. The fuzzy measure of comparison F Comp ( A, B ) = F Sim ( A,B ) – (K-1) , where K is the fuzzy measure of symmetry breaking of conditions previously defined by us from the fuzzy subsethood theorem and measured in fuzzy cardinality. The number 1 is the value of K for every exchange of fuzzy sets as points in the unit hypercube probability space. The resulting fuzzy prediction measure F Comp ( A,B )/F Sim ( A,B ) is multiplied by the risk reduction for the average patient of the Caprie study to find the predicted risk reduction for the clinical patient at hand.

The following definitions are those used in the computation of our fuzzy measure of prediction:
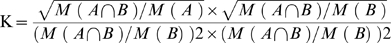
Where K is the fuzzy measure of breaking symmetry of conditions. Like K, 1/K changes symmetry of conditions in the sense that it restores them to a degree. The measures K and 1/K characterize the transformation of fuzzy set as point A to fuzzy set as point B in the unit hypercube. Transformation is another word for “changes into” or comparison. It involves the action of element value change and symmetry of conditions change.

F Sim (A,B) , F Comp (A,B ) and F Pred (A,B ) are already defined in the above discussion. In the F Comp ( A,B ) expression, ( K-1) stands for the subtraction of all change in symmetry of conditions minus that change which does not take place in the probability space within the unit hypercube.

E Sim ( A , B ) stands for the fuzzy entropy of similarity of fuzzy sets as points A and B. It is a measure of the indistinguishability or fuzzy equality of F Sim ( A , B) and F Comp ( A , B ). It is defined by the fuzzy entropy theorem. It is otherwise expressed as E ( F Sim ( A, B ), F Comp ( A, B ). [7]

In this paper we do not use this measure. It is another way of comparing the similarity of fuzzy sets as points A and B when different conditions are not accounted for ( F Sim ( A,B ) and when they are accounted for ( F Comp ( A , B )). [7] It is a useful measure for comparing two patient groups during the conduct of a clinical trial using fuzzy measures of comparison. [5] It is also useful for comparing the different states of one patient over time at serial measures.

E Symm ( A , B ) stands for the entropy of symmetry, and it is another way of measuring the degree to which the breaking and restoration of symmetry of conditions are equal. It is otherwise expressed as E ( K , 1/K ). This measure is not used in this paper. It is also a useful measure when comparing patients and controls in a clinical trial. It is also useful for comparing the different states of one patient as they are measured at different points over time.

## References

[1] Zadeh LA (2006). Generalized theory of uncertainty ( GTU) –principle concepts and ideas.. Computational Statistics and Data Analysis.

[2] Zadeh LA (1965). Fuzzy sets.. Information & Control.

[3] Helgason CM, Jobe TH (2007). Stroke is a dynamic process best captured using a fuzzy logic based scientific approach to information and causation.. IC Med ( TSI Press).

[4] Helgason CM, Jobe TH (2003). Perception based reasoning and fuzzy cardinality provide direct measures of causality sensitive to initial conditions of the individual stroke patient.. International journal for computational cognition.

[5] Helgason CM, Jobe TH (2007). Fuzzy measures of symmetry breaking of conditions, similarity and comparison. Non statistical information for the single patient.. Open Access Cybernetics & Systemics Journal ( Bentham Science).

[6] Nickman NA, Biskupiak J, Creekmore F, Shah H, Brixner DI (2007). Antiplatelet medication management in patients hospitalized with ischemic stroke.. American Journal Health Syst. Pharm.

[7] Kosko B (1993). Neural networks and fuzzy systems. A dynamical approach to machine intelligence..

[8] Helgason CM, Jobe TH (2005). Fuzzy logic and continuous cellular automata in warfarin dosing of stroke patients.. Current Treatment Options in Cardiovascular Medicine.

[9] Gill S, Loprinzi CL, Sargent DJ, Thome SD, Alberts SR (2004). Pooled analysis of fluoruracil-based adjuvant therapy for Stage II and III colon cancer: Who benefits and by how much?. Clin Oncol.

[10] Gorelick PB, Richardson D, Kelly M, Ruland S, Hung E, for the Aftrican American Antiplatelet Stroke Prevention Study ( AAASPS) (2003). Aspirin and ticlopidine for prevention of recurrent stroke in black patients. A randomized trial.. JAMA.

